# Coupled Finite Element-Finite Volume Multi-Physics Analysis of MEMS Electrothermal Actuators

**DOI:** 10.3390/mi13010008

**Published:** 2021-12-22

**Authors:** Thomas Sciberras, Marija Demicoli, Ivan Grech, Bertram Mallia, Pierluigi Mollicone, Nicholas Sammut

**Affiliations:** 1 Department of Mechanical Engineering, Faculty of Engineering, University of Malta, MSD 2080 Msida, Malta; pierluigi.mollicone@um.edu.mt; 2 Institute for Sustainable Energy, University of Malta, MXK 1531 Marsaxlokk, Malta; marija.demicoli@um.edu.mt; 3 Department of Microelectronics and Nanoelectronics, Faculty of Information and Communications Technology, University of Malta, MSD 2080 Msida, Malta; ivan.grech@um.edu.mt (I.G.); nicholas.sammut@um.edu.mt (N.S.); 4 Department of Metallurgy and Materials Engineering, Faculty of Engineering, University of Malta, MSD 2080 Msida, Malta; bertram.mallia@um.edu.mt

**Keywords:** microelectromechanical systems (MEMS), electrothermal actuator (ETA), V-shaped ETA, multi-physics, fluid–structure interaction, convection, finite element, finite volume, numerical analysis

## Abstract

Microelectromechanical systems (MEMS) are the instruments of choice for high-precision manipulation and sensing processes at the microscale. They are, therefore, a subject of interest in many leading industrial and academic research sectors owing to their superior potential in applications requiring extreme precision, as well as in their use as a scalable device. Certain applications tend to require a MEMS device to function with low operational temperatures, as well as within fully immersed conditions in various media and with different flow parameters. This study made use of a V-shaped electrothermal actuator to demonstrate a novel, state-of-the-art numerical methodology with a two-way coupled analysis. This methodology included the effects of fluid–structure interaction between the MEMS device and its surrounding fluid and may be used by MEMS design engineers and analysts at the design stages of their devices for a more robust product. Throughout this study, a thermal–electric finite element model was strongly coupled to a finite volume model to incorporate the spatially varying cooling effects of the surrounding fluid (still air) onto the V-shaped electrothermal device during steady-state operation. The methodology was compared to already established and accepted analysis methods for MEMS electrothermal actuators in still air. The maximum device temperatures for input voltages ranging from 0 V to 10 V were assessed. During the postprocessing routine of the two-way electrothermal actuator coupled analysis, a spatially-varying heat transfer coefficient was evident, the magnitude of which was orders of magnitude larger than what is typically applied to macro-objects operating in similar environmental conditions. The latter phenomenon was correlated with similar findings in the literature.

## 1. Introduction

MEMS’ scalability allows for innovation within niche engineering applications that require small-scale manipulators, for instance, micro-manipulation activities or biological applications, such as red blood cell mechanical response characterisation [1]. MEMS actuators may be activated by several stimuli, including electro-thermal [1,2,3,4], electrostatic [5,6], pneumatic [7], and piezoelectric [8] stimuli. The parameters of the selected stimulus need to be carefully pre-determined and calibrated for the specific MEMS, as well as for its desired function. In the case of actuators, the output is mechanical (motion) in nature and this phenomenon is well-exploited, as may be seen in numerous state-of-the-art devices reported in the literature [9,10,11,12].

Reliable performance prediction is essential in the initial design and optimisation loops of MEMS devices and, as such, simulation tools are key within the MEMS actuator design workflow. This work aimed at presenting a robust simulation methodology that may be adopted during the design phases of electrothermally activated MEMS devices. The capabilities of this methodology are vast and allow designers to model their structures in the presence of any fluid and ambient conditions, and, in turn, numerically predict the product’s thermal and mechanical output. For instance, in the case of MEMS devices to be used for biomedical applications, such as cell characterisation tools where ambient and operational temperatures are critical to the test procedure [1,2], this methodology also allows the user to determine whether the ambient fluid in the vicinity of the test specimen is increasing in temperature, resulting in a detriment to the test specimen and hence the procedure in general.

## 2. Electrothermal MEMS Actuating Principles and Fabrication Process Overview

Electrothermal actuators (ETAs) are devices that are instigated by the user to produce internal heat generation as an activation mechanism. They are composed of an electrically and thermally conductive material that generates internal heat with the flow of a controlled electric current. This phenomenon is known as the Joule heating effect [13]. The internal heat generation causes localised heating of specifically designed structures/elements resulting in thermal expansion. The design is typically tailored in such a way to obtain required translations and device function in accordance with the spatial temperature distribution within the device.

**Figure 1:**
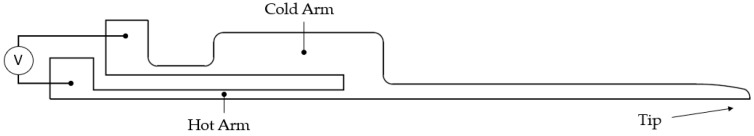
A 2-dimensional plan view representation of a typical U-shaped electrothermal actuator (ETA) (only half of the mechanism is shown).

One very popular MEMS ETA is the U-shaped or ‘hot-and-cold-arm’ device [1,2,3,13]. As indicated in Figure 1, such a structure is composed of two thermally activated members whereby their geometry is characterised by different aspect ratios, the purpose of which is for them to reach different temperatures when activated. Note that the member denoted as hot arm is more slender than the cold arm and, as their name implies, the hot arm reaches much higher temperatures than the cold arm. This leads to the hot arm expanding more than its cold arm counterpart since thermal expansion is directly proportional to temperature and, in this way, a rotation about the flexure member is generated. Displacement amplification means are typical features implemented in such ETAs, which are, in turn, typically characterised by large displacements at their tip for relatively low electrical power inputs [4].

**Figure 2:**
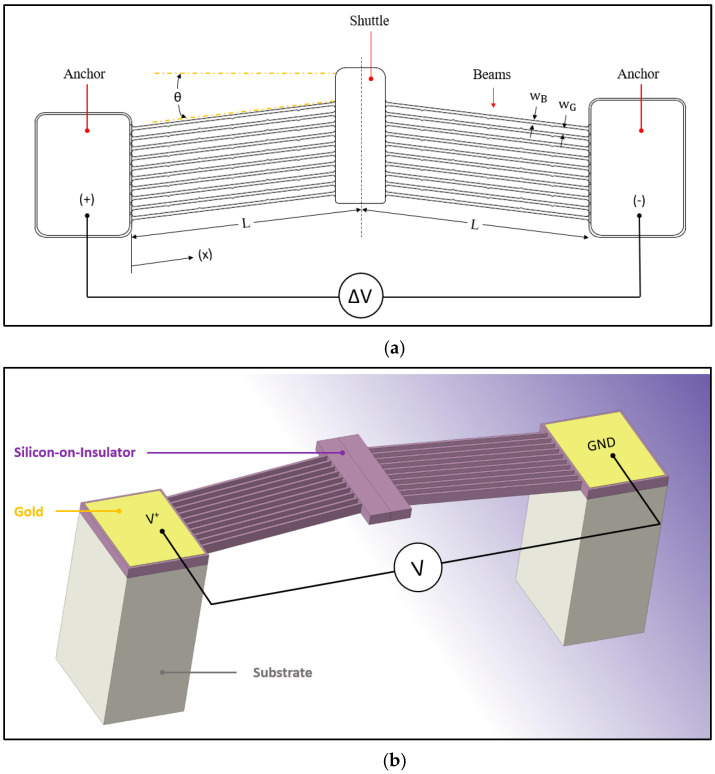
(**a**) A 2-dimensional plan representation of a V-shaped ETA. (**b**) A 3-dimensional view of a V-shaped ETA. Note that the silicon oxide insulating layer (between the silicon-on-insulator (SOI) and the substrate) is excluded from the schematic and simulations.

Another interesting and popular ETA mechanism is the V-shaped ETA, often also referred to as the Chevron-type ETA [14]. In its simplest form, the V-shaped ETA is composed of a series of beams interconnected by two anchors and a central shuttle (Figure 2). The electric potential (V) is applied between the two anchors, which are mechanically fixed structures that allow for virtually no translation and/or rotation. The beams and the shuttle that are suspended act as moving/dynamic elements. Contrary to the hot and cold arm design, the V-shaped ETA does not depend on differential heating to render a displacement but rather on global joule heating of the beams. The structure may be perceived as a fixed-fixed structure whereby when the beams expand, the tendency would be for them to buckle. For this reason, a pre-bend angle (θ) is applied to the beams to facilitate the in-plane displacement in the required direction.

**Figure 3:**
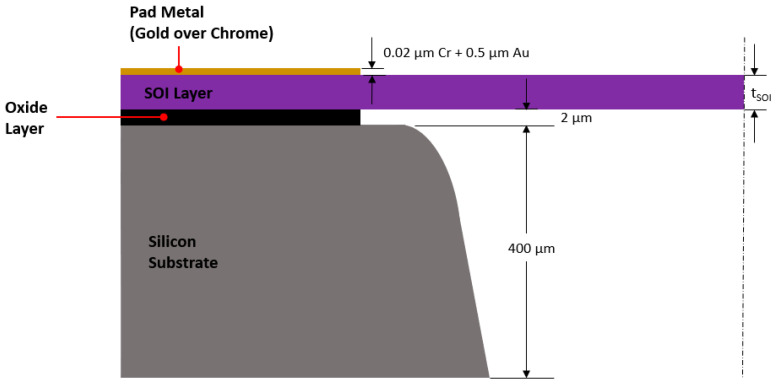
Section view of a SOIMUMPS structure exhibiting a process stack (not to scale). Figure based on procedures described in [3,15].

This work focused on a V-shaped ETA, in particular, one manufactured using the SOIMUMPS^TM^ (Silicon-on-Insulator Multi-User MEMS Processes) micromachining process. The SOIMUMPS stack schematic is illustrated in Figure 3. The reader is referred to [15] for a description of the process and design rules. The geometric parameters associated with the structure under analysis in this work may be seen in Table 1 and are duly illustrated in Figure 2a and Figure 3. This design concept was the candidate of choice for assessment due to its simplicity, popularity, and potential in various engineering sectors. The V-Shaped ETA may be combined with various secondary amplification mechanisms that, as such, make it a versatile driver for multiple applications in microgripper use [16,17]. In subsequent analyses, nominal dimensions, as tabulated in Table 1, were considered. All process-induced irregularities and tolerances are omitted.

## 3. Silicon-on-Insulator Mechanical and Thermal Properties

The SOI microstructure is monocrystalline in nature and often termed single-crystal silicon (SCS). Its mechanical properties exhibit anisotropy due to cubic symmetry, and therefore, the elasticity parameters differ depending on the load direction relative to the crystal orientation [18]. Furthermore, the electrical and thermal properties of SOI silicon are significantly temperature dependent. The said properties’ temperature dependency was excluded from this study as the goal for future work is to devise a very low-power MEMS ETA for a biomedical application. In this case, the device’s temperature sensitivity is expected to be so high (in that the device actuates with a minimal temperature increase) that the properties’ temperature dependency shall no longer be a contributing variable. It is advisable that unless justified, all the temperature and directional dependencies of the parameters constituting the physical nature of the material are considered. The material properties considered in this study were extracted from [3,14] and are given in Table 2.

## 4. Analytical Modelling

MEMS analyses are typically multi-physics in nature in that they include the effects of multiple physical phenomena coupled within the boundaries of the same analysis. In the case of a MEMS ETA, the stimulus is an electrical potential/current, which, in turn, invokes thermal heat generation and hence expansion, the end effect of which is then a mechanical strain. This mechanical strain is then often used to perform a function of sorts, be it micro-object manipulation [4], or characterisation [16].

To date, a good amount of MEMS analysts postulate that heat loss by convection is either negligible and therefore unaccounted for in their analyses [19], or that the heat lost by convection may be assumed as a boundary condition with a constant convection coefficient irrespective of the spatial distribution of surface temperature [6]. Another theory is that the heat lost by convection on the microscale tends to be negligible compared to that lost by conduction to the substrate [20,21,22,23,24,25,26]. Aravind et al. [14], proposed that the temperature distribution within a V-shaped ETA may be calculated using Equation (1):

(1) $$T\left(x\right)\;={\;C}_{1}e^{\mathrm{mx}}+{\;C}_{2}e^{-\mathrm{mx}}+\frac{g_{m}A_{C}}{h_{\mathrm{air}}\cdot p}+{\;T}_{\infty }$$

where T(x) is the temperature as a function of distance along the beam (x); $C_{1}$ and $C_{2}$ are constants of integration; m is what is referred to as the ‘fin parameter,’ given by Equation (2); $g_{m}$ is the ohmic power generated per unit volume given by Equation (3); $A_{C}$ is the cross-sectional area given by $A_{C}={\;w}_{B}\cdot t_{\mathrm{SOI}}$; $h_{\mathrm{air}}$ is the convection coefficient in air; p is the cross-sectional perimeter; and $T_{\infty }$ is the ambient, steady-state temperature.

(2) m =hair·pkSOI·AC

(3) $$g_{m}=\frac{V^{2}}{2R\cdot L\cdot A_{C}}$$

where $k_{\mathrm{SOI}}$ is the thermal conductivity of SOI, V is the applied voltage, and R is the resistance of the SOI structure given in terms of the SOI resistivity ρ in Equation (4):

(4) $$R\;=\frac{\rho \cdot 2L}{A_{C}}$$

The constants $C_{1}\;$and $C_{2}$ may be determined by applying boundary conditions as follows: $T\left(x\right)\;={\;T}_{\infty }$ at x = 0 µm and at x = 2L.

Substituting Equations (2) to (4) into Equation (1) and applying the above boundary conditions,$C_{1}$ and $C_{2}$ were found to be:

(5) $$C_{1}=\left(\frac{g_{m}A_{C}}{h_{\mathrm{air}}\cdot p}\right)\times \left[\left(\frac{\left(1-e^{2\mathrm{mL}}\right)}{\left(e^{-2\mathrm{mL}}-e^{2\mathrm{mL}}\right)}\right)-1\right]$$

(6) $$C_{2}=-\left(\frac{g_{m}A_{C}}{h_{\mathrm{air}}\cdot p}\right)\times \left(\frac{\left(1-e^{2\mathrm{mL}}\right)}{\left(e^{-2\mathrm{mL}}-e^{2\mathrm{mL}}\right)}\right)$$

Despite not being mentioned in [14], it is apparent that Equation (1) is derived from the differential equation as given by Equation (7):

(7) $$\frac{\partial^{2}T}{\partial x^{2}}-\frac{h_{\mathrm{air}}\cdot p}{k_{\mathrm{SOI}}\cdot A_{C}}\left[T\left(x\right)-T_{\infty }\right]\;+\;\frac{g_{m}A_{C}}{k_{\mathrm{SOI}}\cdot A_{C}}=0$$

Although it is advisable to determine the uniqueness and existence of such solutions as described by Equation (1), the current work did not perform such an assessment, and therefore, the presence/potential of “ghost solutions” is currently unclear [27].

All of the previous assumptions may be valid and/or reasonable when designing a MEMS ETA to function in simpler, ideal conditions and with environmental loading, such as exposure in still air. However, this may not always be the case for all MEMS devices. There exist scenarios whereby frictional, pressure, and thermal loads are imparted onto the device by the surrounding fluid. Consider, for instance, a MEMS device whose intended function is in vitro flow measurements [28] or gas flow sensors [29]. MEMS devices in both of these applications broadly require a thermal input from their surroundings to perform their intended function. In such cases, the above-mentioned assumptions may prove to be unsuitable during the design stages of the MEMS device, and therefore, this work sets out to propose a higher accuracy and more holistic approach that may be used by analysts during their design stages in an attempt to promote a more robust device.

## 5. Numerical Modeling Methodology

This section describes two numerical modelling methodologies adopted in this work: the state-of-the-art, fully coupled numerical model that includes fluid–structure interaction and an already accepted sequentially coupled numerical method. All methods, including the analytical model presented in the previous section, are then compared.

### 5.1. Sequential Coupling Methodology via the Finite Element Method

When implementing a sequentially coupled finite element analysis, the implication is that the upstream analysis (the first one that is solved in a paired sequence of numerical analyses) is not influenced by the downstream one, that is, the upstream numerical analysis solves for a given model with certain loads and boundary conditions independently from its downstream successor. The downstream module on the other hand imports the loads from the upstream analysis and uses them to perform the required calculations without influencing the upstream module in any way. Therefore, computational communication between the two analyses is unidirectional and hence termed ‘sequential’.

**Figure 4:**
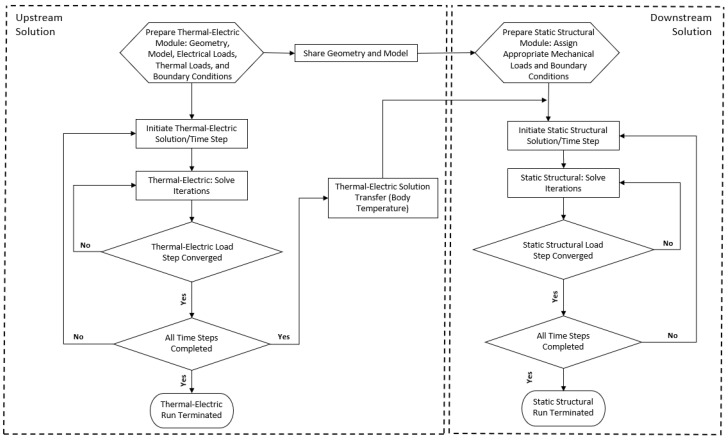
Sequential-coupling process flow execution. The upstream thermal-electric module (**left**) is initiated by the definition of the problem geometry and its discretisation, together with the assignment of loads (thermal and/or electric) and boundary conditions (typically thermal). Following the model definition, the method proceeds to solving all iterations and load steps. Once the solution has converged, the upstream run is terminated. The geometry and model from the upstream solution are shared with the downstream structural module (**right**), where structural loads and boundary conditions are applied as necessary. The body temperature as calculated in the upstream model is also added to the downstream as a thermal load. The downstream module proceeds to solving all iterations and load steps and once the solution has converged, the downstream run is terminated, following which, the evaluation of thermal stresses and strains is possible. All sequentially coupled analyses are implemented in Ansys^®^ Academic Research Mechanical, Release 21.1.

Consider a MEMS ETA in which electrically induced internal heat generation is used to obtain a prescribed mechanical output. A conventional way of solving for such a scenario is to prepare the upstream simulation environment, which allows for geometry discretisation into finite elements whose definition supports both electrical and thermal degrees of freedom. In so doing, both thermal and electrical loads, as well as boundary conditions, can be applied as required by the product specifications. In itself, this amounts to a coupled field analysis since it is made up of and solves for both the mentioned domains, namely, thermal and electrical, within the confines of the same calculation iteration. In such a numerical study, internal heat generation as a result of the electrical load is calculated and this may be used by subsequent analyses. Next, a downstream module, which receives the simulation data (body temperature as a result of internal heat generation in the context of this study) from its predecessor, is required. Here, the model must be discretised into finite elements that have structural degrees of freedom to simulate the nature of actuation (deformation as a result of thermal strain) generated via the internal heat generation. Postprocessing routines also typically involve structural stress analysis, the likelihood of buckling and, if required, analysis of forces exerted onto third-party objects. This sequence of events is described by the flowchart in Figure 4.

#### Sequential Coupling—Model Setup

**Figure 5:**
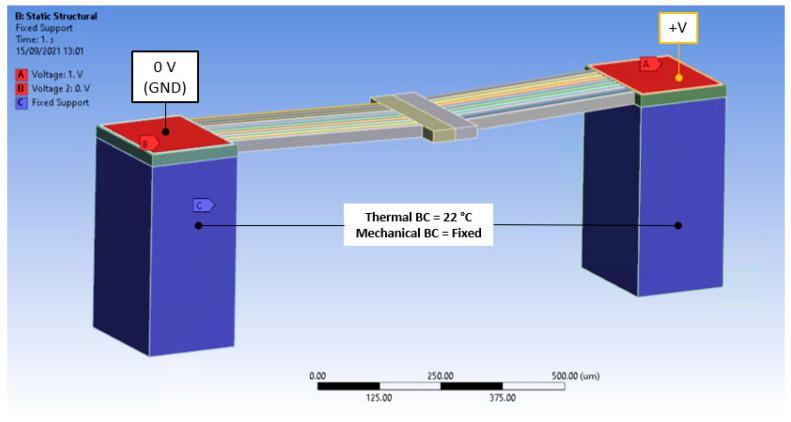
The basis of the thermal-electric model setup within the finite element solver, in this case, ANSYS Mechanical. This setup is applicable to the finite element model of both numerical methodologies presented within this work.

With reference to Figure 5, the geometric model as set up in ANSYS Mechanical^TM^ includes the SOI layer, the gold layer, and the substrate. The reason why the substrate was added to this study is primarily to utilise it as the source of the thermal boundary condition (heat sink), as well as the mechanical fixation support. Furthermore, it is an especially important element for the consequent two-way coupling analysis (since it allows for a real-life representation of the volume of air beneath the suspended SOI structure), and therefore, serves as a necessary direct geometrical representation for comparison between the two analyses. With reference to Figure 3 and Figure 5, the chromium under-strike and the silicon-oxide layers were omitted from the study for two main reasons: first, their influence on the overall performance was deemed negligible, and second, removing them brought about the added benefit of relieving some computational effort from the simulation. As seen in the upcoming sections, the reason for removing the oxide layer from the simulation was essentially because the substrate layers under the SOI anchors were thermally fixed at a specific temperature throughout the duration of the analysis. The chromium layer, on the other hand, was omitted due to the fact that its thickness was negligible compared to that of the gold (refer to Figure 3) and the electrical potential was assumed to reach the SOI anchors uniformly without being affected by temperature.

This study only considered ‘steady-state’ operation in that the temporal/transient effects of both the current drawn and temperature increase within the semiconductor were not analysed. The reasoning here was not only that the device’s performance of interest was, in fact, in a steady state but also that no viscous and/or turbulent effects were expected to interfere with the device’s function. Note that this was because the scenario under investigation consisted of a low-viscosity medium (air) that was not imparting any major pressure effects and/or forced convection. The geometry was discretised with higher-order, 20-node brick-type elements with two degrees of freedom per node, namely, temperature and voltage. No contact-related elements were used, but rather nodes at areas of contact were shared between adjacent bodies in order to reduce the model complexity. Higher-order, quadratic, 20-node brick-type elements with structural degrees of freedom (i.e., displacement) were then used in the subsequent structural finite element model presented in Section 5.2.

For the MEMS ETA, as described in previous sections and as illustrated in Figure 5, the loads and boundary conditions adopted for this analysis were as follows:

In the thermal-electric (upstream) analysis:

i. A potential difference (V) was applied between the gold components (at the anchor sites) by setting one at the positive potential (V^+^) and the other constantly grounded at 0 V. The positive potential was ramped in a series of quasi-static load steps from 0 V to 10 V in steps of 1 V.
ii. All the substrate volume was thermally fixed at a constant temperature of 22 °C. It was assumed that the substrate, given its bulk form in comparison to the suspended structure, acted as a perfect heat sink and maintained the initial, ambient temperature throughout the process.
iii. A constant convection coefficient of $h_{\mathrm{air}}$ = 25 pW/µm^2^·K acting on all exposed surfaces was applied as a boundary condition with an ambient temperature (T_∞_) of 22 °C. This value was chosen to benchmark with an analytical model presented in [14].

In the static structural (downstream) analysis:

i. The only load applied in this analysis was the body temperature from the upstream component. No other external loads were added as the device was being studied as a stand-alone module.
ii. As a mechanical boundary condition, all the substrate volume was assumed as being mechanically fixed, that is, no translations were allowed.

The thermal-electric analysis solved for the thermal-electric behaviour, including Joule heating, using Equation (8) [30]:

(8) $$\left[\begin{array}{cc} \left[C^{t}\right] & \left[0\right]\\ \left[0\right] & \left[C^{V}\right] \end{array}\right]\left\{\begin{array}{c} \left\{\overset{\cdot }{T}\right\}\\ \left\{\overset{\cdot }{V}\right\} \end{array}\right\}\;+\;\left[\begin{array}{cc} \left[K^{t}\right] & \left[0\right]\\ \left[K^{\mathrm{Vt}}\right] & \left[K^{V}\right] \end{array}\right]\left\{\begin{array}{c} \left\{T\right\}\\ \left\{V\right\} \end{array}\right\}\;=\;\left\{\begin{array}{c} \left\{Q\right\}+\left\{Q^{P}\right\}\\ \left\{I\right\} \end{array}\right\}\;$$

where $\left[C^{t}\right]$ is the element-specific heat matrix, $\left[C^{V}\right]$ is the element dielectric permittivity coefficient matrix, $\left\{\overset{\cdot }{T}\right\}$ is the time derivative of temperature, $\left\{\overset{\cdot }{V}\right\}$ is the time derivative of voltage, $\left[K^{t}\right]$ is the element thermal conductivity matrix, $\left[K^{\mathrm{Vt}}\right]$ is the element Seebeck coefficient coupling matrix, $\left[K^{V}\right]$ is the element electrical conductivity coefficient matrix, $\left\{T\right\}$ is the temperature, $\left\{V\right\}$ is the voltage, $\left\{Q\right\}$ is the sum of the element heat generation and element convection, $\left\{Q^{P}\right\}$ is the element Peltier heat load vector, and $\left\{I\right\}$ is the vector of the nodal current. The static structural analysis on the other hand solved for the thermal strain vector with the use of Equation (9) [30]:

(9) $$\left\{\varepsilon \mathrm{th}\right\}\;=\Delta T{\left[\begin{array}{cccccc} \alpha_{x}^{\mathrm{se}} & \alpha_{y}^{\mathrm{se}} & \alpha_{z}^{\mathrm{se}} & 0 & 0 & 0 \end{array}\right]}^{T}$$

where εth is the thermally induced strain vector; ΔT is the change in temperature from the assigned reference temperature (the reference temperature being the zero-strain temperature of the material); and $\alpha_{x}^{\mathrm{se}}$, $\alpha_{y}^{\mathrm{se}}$, and $\alpha_{z}^{\mathrm{se}}$ are coefficients of thermal expansion in the x-, y-, and z-directions, respectively.

### 5.2. Two-Way System Coupling

**Figure 6:**
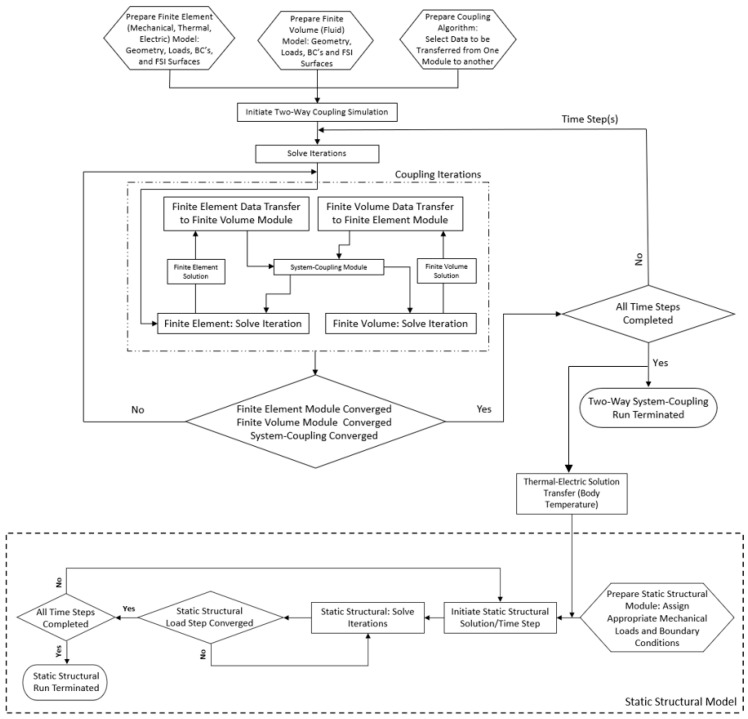
Two-way system coupling—process flow execution. The process flow is initiated by the definition of both finite elements (thermal-electric); finite volume geometries, loads, and boundary conditions; and the interfacing surfaces between the pair. At this stage, the data transfer sets between the finite element and finite volume modules are selected (in this study, data transfer sets during the fully coupled analysis were purely thermal). The solution is then initiated whereby the finite element and finite volume solvers calculate iterations simultaneously, with data transfer between the two models being managed by the coupling algorithm. Once all iterations and load steps have converged, the coupled analysis is terminated. Another finite element model (denoted by the ‘static structural model’) is prepared, including any additionally required structural loads and boundary conditions. The body temperature results as obtained by the coupled analysis are then sequentially transferred as a thermal load to this structural finite element model for the calculation of thermal stresses and strains. All coupled analyses are implemented in Ansys^®^ Academic Research Mechanical, Release 21.1 and Ansys^®^ Academic Research Fluent, Release 21.1.

When a more stringent and holistic multiphysics analysis involving fluid–structure interaction (FSI) is required, a ‘two-way system coupling’ is a state-of-the-art numerical methodology with the potential of catering for all FSI influences collectively and in sync [31]. In brief, this method involves linking two numerical models: one is a model composed of finite elements (such as static or transient structural, steady-state or transient thermal, or thermal-electric) and the other is a finite volume model. The solution of both is found through a ‘system coupling’ algorithm, which is simultaneously and iteratively solved. Therefore, at each iteration, both models communicate with one another via ‘data transfer’ at the FSI interface and are converged in parallel by taking into account the influence of one on the other. Data transfer can have many forms, including force, displacement, pressure, surface temperature, heat transfer coefficient, and heat flow. A flow chart representing the solution execution is shown in Figure 6. It is important to note that after the ‘run termination,’ the analyst may sequentially couple the two-way system coupling solution to post-processing routines or even to another mechanical analysis. This application is discussed and shown in the following sections.

In the previous analysis with the analytical and sequential coupling numerical model (Section 5.1), the model setup consisted of the V-shaped ETA operating at a steady state in air at voltages ranging from 0–10 V. A two-way system coupling replicating the same conditions was set up, this time including the FSI between the fluid medium (air) and the MEMS ETA.

The finite element model was implemented using the steady-state thermal-electric module from ANSYS Mechanical^TM^, while the finite volume model was implemented in ANSYS Fluent^®^. The modules were strongly coupled using the ANSYS System Coupling^TM^ feature. The reader should recall that the work in previous sections dealt solely with the steady state due to the expectation of no turbulent or viscous effects affecting the device structure and function. This assumption was also a vital piece of information in this FSI assessment since this assumption broadened to include the fact that the effect of the fluid on the device performance was only due to its effect on the thermal function, that is, the prime influence from the fluid on the overall device function was its cooling effect. It was for this reason that the thermal-electric module was coupled to the ANSYS Fluent module via system coupling, after which, a static structural module was then sequentially coupled to the resulting (post successful system coupling with ANSYS Fluent) thermal-electric solution for mechanical function analysis.

#### 5.2.1. Model Setup—Finite Element

The thermal-electric finite element model was set up in a similar manner as discussed in Section 5.1 whereby the loads and boundary conditions listed in a.i. and a.ii were applied in the same manner. The boundary condition a.iii (convection boundary condition), on the other hand, was not included this time around since the heat flux from the device was enabled and computed by the finite volume analysis via the system-coupling algorithm. One additional boundary condition within the thermal-electric analysis was the assignment of the FSI surfaces. This was in essence what enabled the simulation and coupling algorithm to identify the geometry (which may either be a set of surfaces or even volumes) of the finite element model where data transfer occurred [30]. The FSI surfaces in the thermal-electric analysis were defined as all the device’s exposed surfaces that made contact with the surrounding fluid.

In the previous analysis with the analytical and sequential coupling numerical model (Section 5.1), the model setup consisted of the V-shaped ETA operating at a steady state in air at voltages ranging from 0–10 V. A two-way system coupling that replicated the same conditions was set up, this time including the FSI between the fluid medium (air) and the MEMS ETA.

#### 5.2.2. Model Setup—Finite Volume

**Figure 7:**
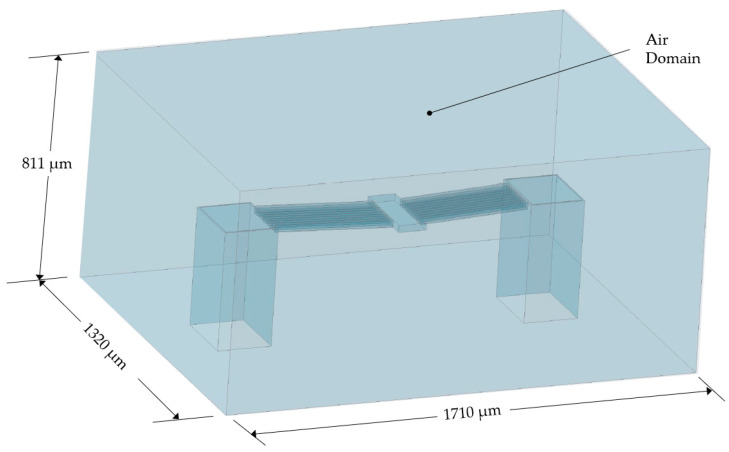
Geometry as prepared for the finite volume model. This geometry was discretised into cells and acted as air in the coupled numerical analysis. Note that from a geometry perspective, the fluid enclosure was modelled as a computer-aided designed ‘solid’ with the V-shaped ETA structure cut away from it. The FSI interface between the finite element and finite volume models was a perfect match at the said cut-out, and this surface set, composing the cut-out, formed the data transfer location. The dimensions are given in micrometres.

The finite volume domain was prepared as shown in Figure 7, whereby the air domain was modelled such that it completely surrounded the MEMS ETA with excess material to allow for heat transfer computations. Hence, the ETA itself was not included in the finite volume as it was included in the finite element environment only. Its geometry was, however, cut out from the air domain in the finite volume and the resulting FSI surfaces formed the location of data transfer during the numerical coupling.

Since the current case study’s computations involved the transfer of thermal degrees of freedom, the finite volume environment was set such that the energy equation, as given by Equation (10), was computed numerically [32].

(10) $$\frac{\partial }{\partial t}\left(\rho \left(e\;+\frac{v^{2}}{2}\right)\right)+\nabla \left(\rho v\left(h+\frac{v^{2}}{2}\right)\right)=\nabla (k_{\mathrm{eff}}\nabla T\;-\underset{j}{\sum }h_{j}\vec{J_{j}}\;+\;{\bar{\tau }}_{\mathrm{eff}}\cdot \vec{v})\;+\;{\;S}_{h}$$

where ρ is the fluid density, e is the internal energy, v is the fluid velocity, h is the fluid enthalpy, $k_{\mathrm{eff}}$ is the effective conductivity, T is the temperature, $h_{j}$ is the portion of enthalpy brought about by specific heat of species ‘j’, $\vec{J_{j}}$ is the diffusion flux of species ‘j,’ ${\bar{\tau }}_{\mathrm{eff}}$ is the stress tensor, $\vec{v}$ is the velocity vector, and $S_{h}$ includes any volumetric heat generation sources and heat sources brought about by chemical reactions. The first two terms on the left-hand side of Equation (10) are the transient term, which shall be omitted from this analysis since only the steady-state is being considered here, and the convection term, respectively. The first two terms on the right-hand side represent energy transfer via conduction and energy transfer via species diffusion, respectively, with the third term on the right-hand side concerning viscous heating. The latter term shall not be considered in this analysis since the current investigation only concerns itself with laminar conditions.

Given that the scenario under investigation involves merely natural convection dominated by buoyancy forces at the FSI interface, coupled with relatively low anticipated temperatures, laminar conditions were assumed within the finite volume, and the fluid (air) was set as an incompressible ideal gas.

As far as boundary conditions were concerned, the surface area at the base of the model was assumed to be a fixed wall at a constant temperature of 22 °C, thus simulating the work surface on which the MEMS was tested. The remaining five extremities of the enclosure were assumed to be pressure outlets at atmospheric pressure (~1 bar) with the allowance of reverse flow, and a re-entry temperature also of 22 °C. The final boundary condition was that the FSI surfaces’ temperature was controlled via the system-coupling module.

#### 5.2.3. Data Transfer at the Fluid–Structure Interface

The variables for data transfer between coupled finite element and finite volume analyses as set up in the system coupling algorithm, together with their associated source and target modules, may be seen in Table 3.

As shown in Table 3, the finite volume was the numerical environment responsible for imparting convective heat transfer, whereby the ‘heat transfer coefficient’ was computed within the finite volume using Equation (11) and then relayed this as the convection coefficient within the finite element environment [33].

(11) heff =q(Twall− Tref)

where $h_{\mathrm{eff}}$ is the total heat transfer coefficient, q is the heat flux per unit area at the surface, $T_{\mathrm{wall}}$ is the temperature of the surface at a particular location, and $T_{\mathrm{ref}}$ is the reference (ambient) temperature. Together with convection-related variables, the finite volume also transferred the ‘near-wall temperature’ to the finite element environment. This was the temperature as calculated in the cells adjacent to the finite elements and was used as the reference temperature ($T_{\mathrm{ref}}$) in Equation (9). The finite element environment, on the other hand, transferred the temperature data at the FSI to the finite volume for it to react accordingly.

Following a successful run termination when all three major components of the numerical model (finite element, finite volume, and system coupling) converged, the resulting thermal-electric results were finally sequentially coupled to a structural analysis, where the structural displacements were then evaluated.

## 6. Results and Discussion

The thermal response of a MEMS ETA to the applied, electrical stimulus is a crucial element of consideration, not only due to it being directly proportional to the MEMS’ main function, that is, displacement, but also owing to their niche applications, such as cell manipulation or micro-object characterisation and manipulation [16]. In view of such sensitive applications, MEMS devices must be devised in such a manner as to not impart any damage to the object being manipulated by overheating gripping arms/features. Furthermore, the MEMS material itself has temperature limitations that cannot be exceeded to avoid either material softening [4], pad metal delamination, or damage of any form [1]. Although the temperatures reached in this study were well within limits, maintaining such criteria was not the focus of this study since the scope was primarily to demonstrate the benefits of a two-way coupling methodology.

**Figure 8:**
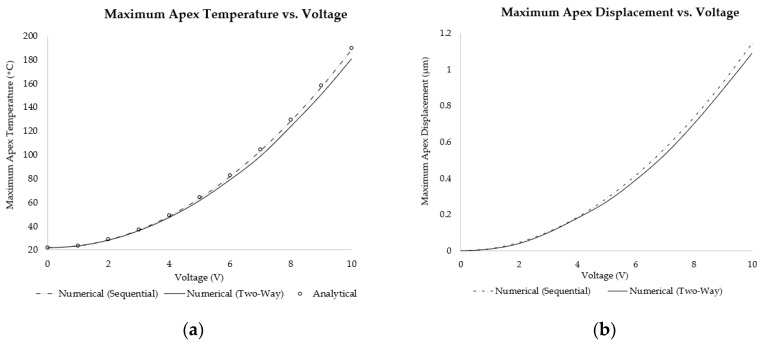
Plots of the (**a**) maximum temperature vs. voltage and (**b**) maximum displacement vs. voltage of the V-shaped ETA using analytical, sequentially coupled numerical and two-way system coupling numerical analyses.

**Figure 9:**
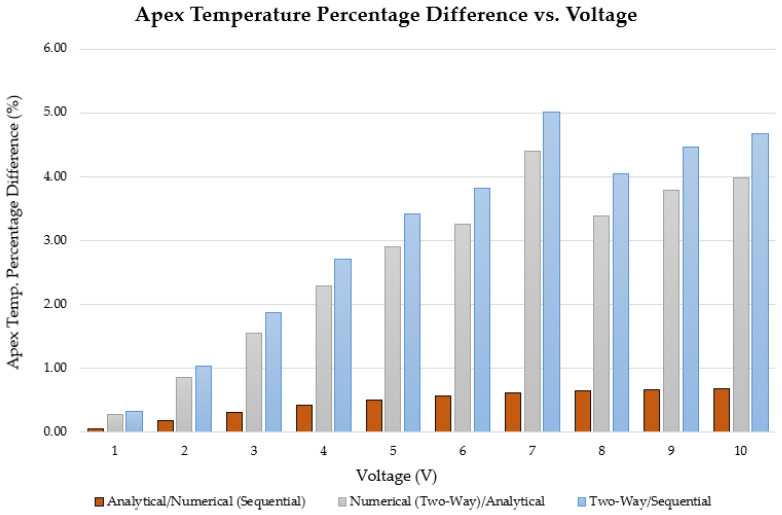
Percentage difference comparison of maximum apex temperatures as obtained via analytical and numerical methodologies. Results from all three methods were compared with one another.

The electro-thermo-mechanical performance of the V-shaped ETA was assessed using the three methodologies described in the previous sections. The primary focus of the results was the thermal and displacement response for voltage inputs ranging from 0 V to 10 V, paying particular attention to the effect of the impact of heat loss via convection. The maximum temperature (which occurs at the apex or centre of the shuttle of the V-shaped ETA) was calculated using all three methodologies, whereas the corresponding maximum apex displacement was only calculated using the sequentially coupled and two-way coupled numerical methods. Plots of the maximum temperature and shuttle displacement versus input voltage, are shown in Figure 8. The analytical results for the maximum apex temperature were computed using Equation (1). Moreover, the percentage difference of the maximum apex temperature as obtained with the three methodologies (analytical, sequential numerical, and numerical two-way coupled) is shown in Figure 9.

Figure 8 and Figure 9 show that the analytical model and the sequentially coupled numerical results matched very well, exhibiting only minor differences. The minor differences exhibited between the two may be attributed purely to the fact that the analytical model did not account for a ‘shuttle’ between the mirrored beams, but merely considered continuous beams of uniform cross-section. The reduction in surface area brought about by the addition of the shuttle may well be the cause of the minute reduction in temperature and hence displacement. However, the results obtained from the two-way system coupling analysis exhibited larger differences in temperature (in the order of approximately 0.3% at an input voltage of 1 V DC to 4% at 10 V DC input) and displacements, with the trend being a diverging one, that is, the larger the voltage, the greater the discrepancy. This was because the two-way coupling methodology took into account the spatial distribution of temperature and used that information to calculate an also spatially varying heat transfer coefficient. Furthermore, the finite volume did not assume a constant air density and hence accounted for the buoyancy-related convective currents at the FSI.

**Figure 10:**
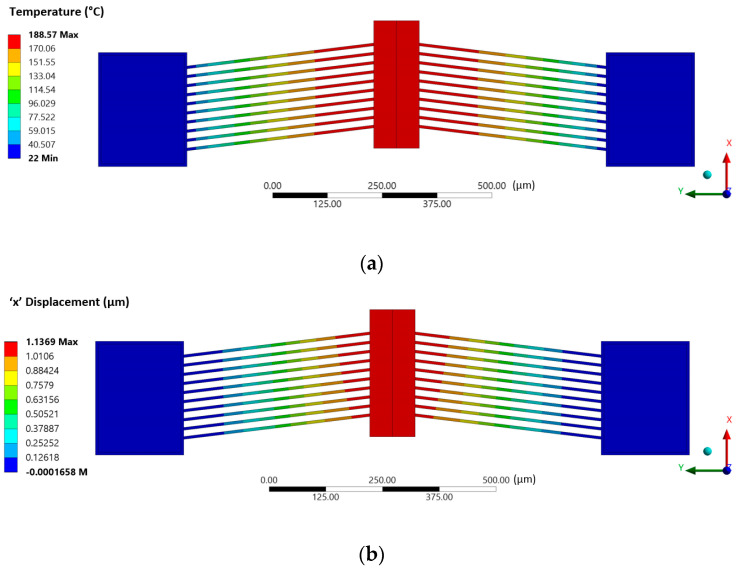
Sequential coupling numerical results of (**a**) temperature distribution and the (**b**) corresponding displacement in the steady-state operation under an applied potential of 10 V DC.

**Figure 11:**
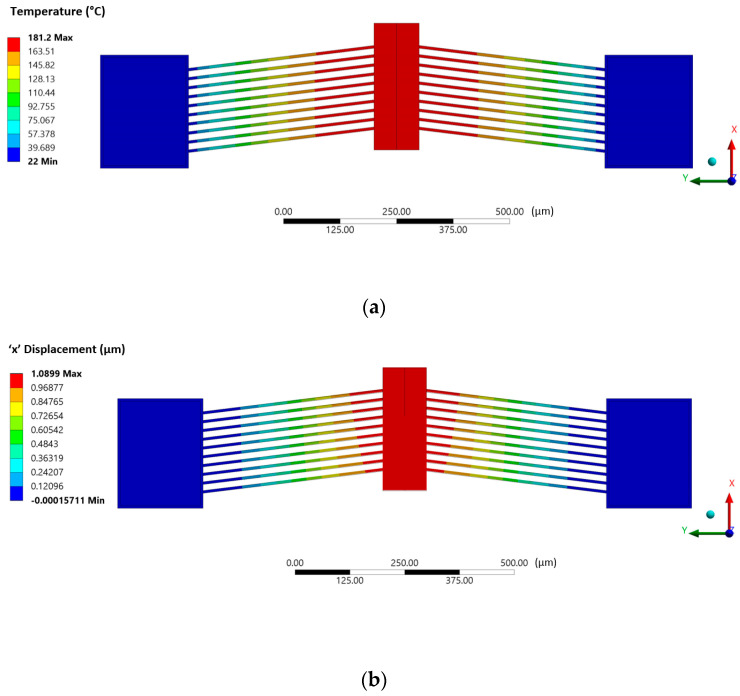

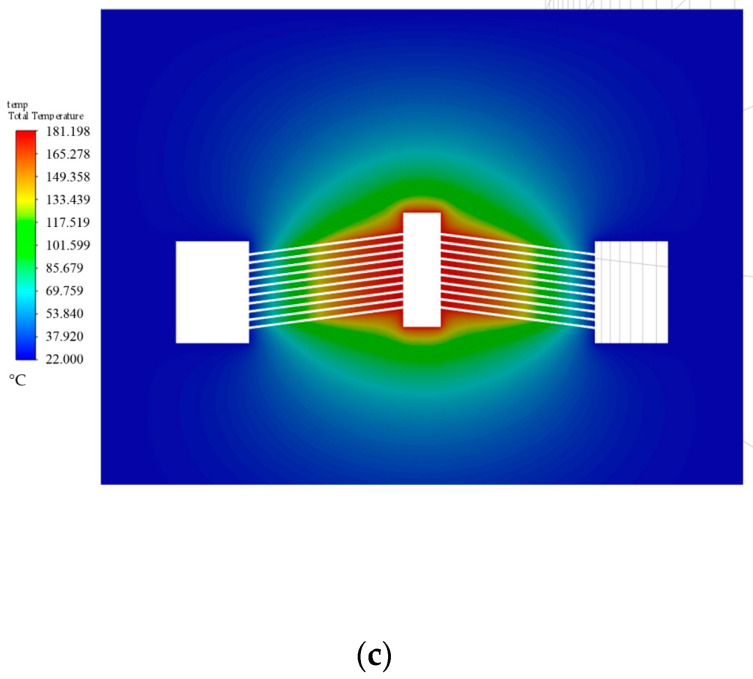
Coupled finite element–finite volume results of the (**a**) temperature distribution within the V-shaped ETA as extracted from the thermal-electric finite element solver (shown in °C), (**b**) displacement of the V-shaped ETA extracted from the structural finite element solver (shown in µm), and (**c**) fluid (air) temperature distribution around the V-shaped ETA extracted from the finite volume solver (shown in °C), all at a 10 V DC input.

A typical spatial temperature distribution in such a MEMS ETA via the sequential-coupling numerical method is shown in Figure 10, together with its corresponding displacement. Similarly, Figure 11 shows the results obtained in both the finite element and finite volume solvers at 10 V input following successful two-way system-coupling computations.

As per typical engineering processes, there exist several MEMS manufacturing process-related factors that may affect the performance of MEMS ETAs. As outlined in [1], such effects may manifest themselves in the form of dimensional variations, as well as mechanical, thermal, and electrical parameter drifts of the functional materials, in particular, the semiconductor. Variations between performance as exhibited in experimental results to those predicted by analytical and numerical models may, however, also be incurred by sub-ideal non-material or structure-related assumptions and boundary conditions at the design stages, such as the omission of convection or the improper assignment of the convection coefficient. Although the contribution of this is currently unquantified, it is somewhat supported by several studies [1,34,35,36] in which it was demonstrated that MEMS ETAs’ numerically predicted displacements exceed the physical/experimental performance. However, a somewhat confusing matter arises when published data conversely exhibits correlations in which the experimental displacement results exceed the numerical ones. This may be seen, for instance, in [1,2,3]. Unlike material property-related discrepancies, whose effects influence all MEMS devices in a similar manner, irrespective of their shape and structural nature, it is anticipated that convection, be it natural or forced, affects the performance of different MEMS devices differently. Considering a U-shaped MEMS ETA similar to those in [1,2,3,4,13], the reader should recall that such devices require an internal thermal gradient between their ‘hot’ and ‘cold’ arms to actuate and that their designation is as such due to their different geometries. Bearing in mind Newton’s law of cooling, shown in Equation (11), it becomes clear that the larger surface area of the cold arm allows for a larger total heat outflow from the said arm via convection in comparison to that of the more slender hot arm, whose surface area is smaller. Countering this argument, however, is the fact that the temperature in the hot arm is higher than that in the cold arm, which also allows for larger heat energy outflow. With reference to the U-shaped ETA in [1], the hot arm is documented to reach temperatures that are approximately 57% higher than that of the cold arm, whereas the cold arm surface area is 370% larger than that of the hot arm, and therefore this poses itself as a potential cause as to why numerical results without active convection computations underestimate the MEMS ETA performance. The same argument does not apply for V-shaped type ETAs since their mechanical output performance is solely determined by a global increase in temperature rather than internal gradients. Therefore, in the case of the data reported in [36], reasons for the slight underprediction of the output displacement of the V-shaped ETA may be threefold: the use of a constant thermal expansion as per the authors’ claims, material properties and geometrical discrepancies, and the accuracy of the measurement method itself.

**Figure 12:**
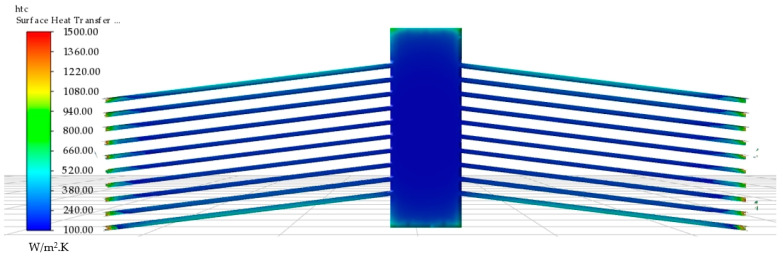
Finite volume numerical evaluation of spatial heat transfer coefficient at the fluid–structure interface. Results displayed are for an applied potential of 4 V. Note that the values in the legend are given in W/m^2^·K, which is equivalent to pW/µm^2^·K.

All the above reinforce the fact that the assumption of a constant heat transfer coefficient that one would apply in macro-modelling (say, 25 pW/µm^2^·K, as in [14]) or a constant heat loss is not an entirely correct assumption and although perhaps not so detrimental under conditions involving merely natural convection in air, it may be an issue with either other media or other medium conditions, such as different flow rates or even highly temperature-sensitive drivers. The claim that a constant heat transfer coefficient (in particular, 25 pW/µm^2^·K, which is typically employed for natural convection in air at the macroscale) is not ideal for microscale devices is also supported by the work undertaken in [37], where it was hypothesised that the convection heat transfer coefficient may in fact reach values of orders of magnitude larger than that for larger-scale macro-objects. With the use of the two-way system coupling methodology, such larger coefficients are captured here. Figure 12 shows the spatial variation of the heat transfer coefficient in this study along the FSI area of interest with a 4 V DC input.

Figure 12 shows the larger coefficients at the ends of the beams towards the substrate. The reason for this relatively sharp increase in the heat transfer coefficient was due to the substrate surfaces being thermally clamped at 22 °C (295.15 K), thus acting as a perfect heat sink at the substrate–SOI interface. Another interesting observation was that the resulting heat transfer coefficient at the four outermost beams was also much higher than the internal ones (approximately 600 pW/µm^2^·K on the outer beams compared to 200 pW/µm^2^·K on the inner beams) owing to the fact that the said four beams were more exposed and hence exhausted more heat to the atmosphere than the internal ones. This was also substantiated by Figure 11c, where it is shown that the temperature gradient around the outer beams was larger than that of the internal ones. This form of active heat transfer coefficient computation was what distinguished the two-way coupled analysis from the sequentially coupled finite element model in which the heat transfer coefficient must be known prior and applied as a boundary condition.

## 7. Conclusions

Throughout the course of this study, the popular V-shaped electrothermal mechanism was chosen as the analysis candidate and was used in particular to demonstrate the state-of-the-art, multi-physics numerical modelling technique based on a fully coupled fluid–structure interaction algorithm. Its benefits relative to conventional methodologies, including analytical and numerical methods assuming bulk/macro component heat transfer coefficients, were highlighted. Although the percentage differences between the evaluated results may not seem large, the assessment case involved relatively low operational temperatures and natural convection in air, whereby both criteria imply minimal cooling effects on the driving mechanism. Another important point to bear in mind is the fact that the primary functions of certain MEMS ETA involve temperature-sensitive operations, which, in turn, prompt designers to model highly temperature-sensitive devices. In such a scenario, accurate modelling of the thermal and hence structural performance is a necessity and the proposed methodology excels at providing analysts with models of superior accuracy.

This research paper has outlined the methodology utilised in both sequential and two-way system coupling numerical techniques, and has provided the reader with in-depth process flows. While analytical and stand-alone finite element techniques have to date demonstrated sufficient robustness for simpler scenarios, the coupled finite element–finite volume methodology offers MEMS design engineers an opportunity to model devices for more complicated conditions involving fluid–structure interaction with confidence. It does, however, come at a price of additional computational expense.

The active heat transfer coefficient computation was also calculated by the coupled analysis, whereby the spatial distribution of the heat transfer coefficient was evident. The difference in heat transfer coefficient at the outermost beams, where the temperature gradient was the largest, compared to the inner beams, was also noted. These characteristics show how the two-way coupled analysis is ideal for an accurate analysis of such MEMS devices. Of particular interest was the seemingly large heat transfer coefficient computed by the coupled finite volume and finite element numerical analysis that corresponded to similar observations in the literature. In view of this, some insight was shed upon potential mechanisms that bring about a degree of error between numerical and experimental outcomes for different ETA mechanisms.

Despite only coupling thermal degrees of freedom at the steady-state operational regime, there exist possibilities of coupling both thermal and structural degrees of freedom between finite element and finite volume systems. Such possibilities shall be the focus of future work, whereby a highly temperature-sensitive device shall be numerically and experimentally validated under different operational parameters.

## Figures and Tables

**Table 1:** Nominal geometric parameters of the MEMS structure (see Figure 2a for annotations).

Parameter	Value
Distance from anchor to centre of shuttle parallel to beams, L (µm)	464
Width of beams, $w_{B}$ (µm)	6
Beam spacing, $w_{G}$ (µm)	10
Pre-bend angle, θ (°)	7
Number of beams per side	10
SOI silicon thickness, t_SOI_ (µm)	25

**Table 2:** Material properties of the SOI and pad metal layers as extracted from [3,14]. The below data was assumed to be at a reference temperature of 22 °C. Note that directions ‘x’ and ‘y’ are in-plane, whereas ‘z’ is out-of-plane.

Property	SOI	Pad Metal
Young’s modulus, E (GPa)	E_x_ = E_y_ = 169, E_z_ = 130	57
Shear modulus, G (GPa)	G_yz_ = G_zx_ = 79.6, G_xy_ = 50.9	N/A
Poisson’s ratio, ν	ν_yz_ = 0.36, ν_zx_ = 0.29, ν_xy_ = 0.064	0.35
Density (g/(cm)^3^)	2.50	19.30
Thermal conductivity, k (W/m·K)	148	297
Electrical resistivity (µΩ.m)	500	2.86 × 10^−2^
Specific heat capacity, c (J/kg·K)	712	128.7
Coefficient of thermal expansion, α (µm/m·K)	2.5	N/A

**Table 3:** Data transfers as set up in two-way fluid–structure interaction simulation.

Data Source	Target Module	Source Variable	Affected Target Variable
Finite Volume	Finite Element	Heat Transfer Coefficient	Convection Coefficient
Finite Volume	Finite Element	Near-Wall Temperature	Convection Reference Temperature
Finite Element	Finite Volume	Temperature	Temperature

## Abbreviations

The following abbreviations are used in this manuscript:

BC	Boundary condition
ETA	Electrothermal actuator
FSI	Fluid–structure interface
GND	Ground
MEMS	Micro-electro-mechanical system
SCS	Single crystal silicon
SOI	Silicon-on-insulator
